# Dystonia in Patients with Spinocerebellar Ataxia 3 - Machado-Joseph disease: An Underestimated Diagnosis?

**DOI:** 10.2174/1874205X01812010041

**Published:** 2018-05-31

**Authors:** Ligia Maria Perrucci Catai, Carlos Henrique Ferreira Camargo, Adriana Moro, Gustavo Ribas, Salmo Raskin, Hélio Afonso Ghizoni Teive

**Affiliations:** 1 Botulinum Toxin Unit, Hospital Universitário, State University of Ponta Grossa, Ponta Grossa, Brazil; 2 Movement Disorders Unit, Neurology Service, Internal Medicine Department, Hospital de Clínicas, Federal University of Paraná, Curitiba, Brazil; 3 Neurology Service, Hospital Universitário, State University of Ponta Grossa, Ponta Grossa, Brazil; 4 Paraná Association for Parkinson’s Disease, Curitiba, Brazil; 5 Group for Advanced Molecular Investigation, Graduate Program in Health Sciences, School of Medicine, Pontifícia Universidade Católica do Paraná, Curitiba, Brazil; 6 Genetika-Centro de Aconselhamento e Laboratório de Genética, Curitiba, Brazil

**Keywords:** Dystonia, Machado-Joseph disease, Spinocerebellar ataxias, Genetic epidemiology, Movement disorders

## Abstract

**Background:**

Spinocerebellar Ataxia type 3 (SCA3) or Machado-Joseph Disease (MJD) is characterized by cerebellar, central and peripheral symptoms, including movement disorders. Dystonia can be classified as hereditary and neurodegenerative when present in SCA3.

**Objective:**

The objective of this study was to evaluate the dystonia characteristics in patients with MJD.

**Method:**

We identified all SCA3 patients with dystonia from the SCA3 HC-UFPR database, between December 2015 and December 2016.Their medical records were reviewed to verify the diagnosis of dystonia and obtain demographic and clinical data. Standardized evaluation was carried out through the classification of Movement Disorders Society of 2013 and Burke Fahn-Marsden scale (BFM).

**Results:**

Amongst the presenting some common characteristics, 381 patients with SCA3, 14 (3.7%) subjects presented dystonia: 5 blepharospasm, 1 cervical dystonia, 3 oromandibular, 3 multifocal and 2 generalized dystonia. Regarding dystonia's subtypes, 71.4% had SCA3 subtype I and 28.6% SCA3 subtype II. The average age of the disease onset was 40±10.7 years; the SCA3 disease duration was 11.86± 6.13 years; the CAG repeat lengths ranged from 75 to 78, and the BFM scores ranged from 1.0 to 40. There was no correlation between the dystonia severity and CAG repeat lengths or the SCA3 clinical evolution.

**Conclusion:**

Dystonia in SCA3 is frequent and displays highly variable clinical profiles and severity grades. Dystonia is therefore a present symptom in SCA3, which may precede the SCA3 classic symptoms. Dystonia diagnosis is yet to be properly recognized within SCA3 patient.

The Spinocerebellar Ataxias (SCAs) correspond to a large group of heterogeneous autosomal dominant neurodegenerative diseases, presenting some common characteristics, such as the presence of ataxia and the degenerative process involving the cerebellum and/or its afferent and efferent connections [1]. Other structures of the nervous system tend to be affected, including the basal ganglia, the brainstem nuclei, pyramidal tracts, posterior colomn in addition to the anterior horn of the spinal cord [2].

SCA3 or Machado-Joseph disease (MJD) is considered as the most common ataxia in the world [3, 4]. This disease is caused by a mutation in the unstable CAG expansion of the gene *ATXN3* at chromosome 14q32.12, with an abnormal amount of repetitions between 56 and 86 [2, 4-6]. It is characterized by a wide phenotypic heterogeneity, most commonly, affected persons in the young-adult year with progressive gait imbalance associated with speech difficulties [4]. With the disease evolution other symptoms may appear: different degrees of ataxia until the use of wheelchairs; dysphagia; ophthalmoplegia; pseudoexoftalmia (bulging eyes*),* peripheral neuropathy; signs of pyramidal tract and movement disorders [2, 5-7]. According to the signs and symptoms present, individuals with SCA3 can be divided into seven distinct subtypes phenotypically [8].

The movement disorders are common in SCA3. Dystonia is a movement disorder characterized by sustained or intermittent muscle contractions causing abnormal and often repetitive postures, movements, or both. The dystonic movements are typically standardized, in torsion, and may be in tremors. Dystonia is often initiated or worsened by voluntary activities and associated with muscle activation extravasation [9]. The similarities among the SCAs and dystonia may have potentially shared molecular pathways, using a gene co-expression network approach [10], nevertheless, it still remain uncertain whether dysfunction of one brain area, combined dysfunction of multiple areas, or abnormal communication between different brain areas leads to dystonia [10].

Even though its relevance is well established, dystonia is not properly identified in daily clinical practice, and it may still today, be neglected in the patients’ routine assessment, including those with SCAs [11]. Therefore, the medical practitioner and researcher’s attention should be centered on the characterization and quantification of the severity of this disease potentially incapacitating. The understanding of the various types of dystonia, as well as their severity, is fundamental for high-quality clinical practice, as well as best performance of their scientific discoveries.

The aim of this study was to evaluate the dystonia characteristics in patients with SCA3.

## 1 METHOD

### 1.1 Patients Selection

Amongst the 381 patients from 190 families with SCA3 registered in the SCA3 HC-UFPR database, patients were selected due to having some type of dystonia. These patients already had a clinical and genetic diagnosis of SCA3, or had a first-degree relative with genetic confirmation of SCA3.

Between December 2015 and December 2016, researchers contacted them in order to participate in this study, which was approved by the Human Research Ethics Committee at Clinical Hospital of Federal University of Paraná.

### 1.2 Clinical Assessment

Their medical records were reviewed to verify the diagnosis of dystonia and obtain demographic and clinical data followed by recent movement disorders assessments through physical examination or video analysis, as well as full access to clinical history either via anamnesis with the patient, family members, caregivers, or by clinical records.

All in personal assessments included clinical history, physical and neurological examination. Dystonia evaluation was based on the classification of Movement Disorders Society (MDS) [9], subtype of dystonia [8] and Burke Fahn Marsden scale (BFM) [12].

Those patients who could not be examined in person, the data were collected through medical records and movement disorders examination videos followed by data confirmation (directly with the patients themselves, family members or caregivers by telephone contact).

The data about the affected family members were collected in the same way.

For the movement disorders examination, we used audiovisual data recorded at the latest face-to-face consultation at HC UFPR. These videos were watched by at least two researchers in a 32-inch screen, each patient's video was presented and evaluated at least twice, making it possible to characterize and classify each case of dystonia.

The classification was performed for each affected patient and family member according the SCA 3 subtype: (1) Type Joseph - Ataxia and parkinsonism; (2) Type Thomas - Ataxia and pyramidal signs; (3) Type Machado - Ataxia and peripheral signals; (4) Parkinsonism; (5) Spastic paraplegia; (6) Pure cerebellar syndrome; (7) Mixed type with ataxia, pyramidal signs and parkinsonism responsive to levodopa [3].

Movement disorders specialist confirmed all this data (CHF, AM or HAT).

The neuroimaging examinations data (computed tomography and/or Magnetic Resonance Imaging (MRI) of the brain) were collected from the last examination recorded in the database. All patients had computed tomography results available, while MRI was only for three of them.

### 1.3 Statistical Analysis

All data were tested for distribution pattern (normal or not). Statistical differences among the groups were determined using the one-sided Student's t-test to normal distributions and the Mann-Whitney test for non-normal distributions. Fisher exact test and Pearson correlation coefficients were used when necessary. All the statistical analysis was performed with the Programs *Office Excel* and *Statistica for Windows,* version 99. Differences were considered significant when p<0.05.

## 2 RESULTS

14 (3.67%) patients had dystonia. The mean age, in years of patients was 40±10.74, and the disease duration (SCA3), in years, was 11.86± 6.13. There was a predominance of males in a ratio of 2.5:1 (Table **1**). Genetic evaluation was performed in eight patients, and CAG expansions ranged between 75 and 78 (Table **1**).

In-person assessment was possible in two patients. Seven patients had no clinical conditions to be present for physical examination, and six patients passed away.

All patients presented gait ataxia, dysarthria and nystagmus. Axial ataxia was found in 10 patients (71.4%) and appendicular ataxia in nine patients (64.3%). concerning the ocular alterations, besides nystagmus 12 patients (85.7%) presented horizontal ophthalmoplegia, ten patients (71.4%) pseudoexoftalmia (*bulging eyes*), four patients slow saccade of eyes movements (Table **2**). Regarding the motor symptoms, three patients presented hyperreflexia, ten patients spasticity, eight patients Babinski sign. Face fasciculation were found in 7 patients while body fasciculation were found only in three others. Scoliosis was observed in four of the patients and *pes cavus* in two. Parkinsonism was found in two patients, no other basal ganglia disorders were found (Table **2**). Cerebellar atrophy was found in neuroimaging examinations of all patients, while trunk atrophy was present in only one.

Concerning patients SCA3 subtypes, ten (71.4%) were subtype 1, four (28.6%) were the subtype 2 (Table **2**). As per patients family members, from a total of 30 subjects, one was subtype 1, eighteen subtype 2, seven subtype 3, two of subtype 4, one of subtype 6 and one of subtype 7. None presented subtype 5 (Table **2**).

Of the total of 14 patients, 5 (35.7%) had focal dystonia type blepharospasm, 3 (21.4%) focal oromandibular dystonia, 1 (7.1%) focal dystonia cervical type, 2 (14.3%) multifocal dystonia in hands, 1 (7.1%) multifocal dystonia in hands and oromandibular and 2 (14.3%) generalized dystonia (Table **3**). Oromandibular dystonia was found in three patients, two with an “open moth pattern” and one with ” close mouth pattern”. Cervical dystonia was a left torticollis. Two patients presented multifocal dystonia in both hands whereas one patient has oromandibular and both hands dystonia (Table **3**). The dystonia severity was measured by BFM scale, the mean score was 9.43± 12.1. The lowest value was 1 in a patient with blepharospasm and the highest was 40 in a patient with generalized dystonia (Table **3**).

There was no significant correlation when the values of the severity of the dystonia were correlated with the number of expansions CAG (r=0.3328 CI 95% -0,8404 to 0.4858, p=0.4206), with the gender of patients (r=0.2838, CI 95% -0,2905 to 0.7078, p=0.3255), with disease duration (r=-0,02852, CI 95% -0,5508 to 0.509, p=0.9229), with the age of onset of the disease (r=95, 95% -0,3301 -0,7324 to 0.2431, p=0.2491). There was also no difference in severity of dystonia when compared to the patients with subtype 1 and subtype 2 (p=0.4345).

## 3 DISCUSSION

Only 14 patients had dystonia, meaning 3.67% of the total of 381 SCA3 patients. This number was lower than those available estimates that vary between 5.5 and 33% [7]. This was probably due to lack of attention to dystonia in the physician’s routine evaluation, or the patient’s difficulty in reporting any complaint that is directly related to it. Steeves *et al.* [13] in a systematic review showed that epidemiological studies adopted different methodologies, resulting in widely different prevalence, probably underestimated. They conclude that the attempt to determine an accurate prevalence of dystonia remains a significant challenge for the health services planning. Although understanding of dystonia has improved in recent years, there is consensus among movement disorders specialists that its diagnosis may be underestimated [14]. According to this study, the primary dystonia is still insufficiently recognized and patients may not receive the correct diagnosis. Williams *et al.* [11] described this same difficulty in a study with 592 subjects with isolated focal dystonia from adult onset, in Ireland, by reinforcing that the prevalence rates of primary dystonia can vary from 30 to 7320 cases per million; and also estimates the prevalence of focal dystonia isolated from adult onset from 20 to 137 cases per million.

Through the patients’ reports and the medical records, we observed that the approach to dystonia was inattentive, therefore therapeutic procedures, when performed, were delayed. Beghi *et al.* [15] investigated the reliability of dystonia diagnosis among neurologists with different levels of professional experience using videos of patients with and without dystonia. Blind raters diagnosed each case. Sensitivity were 95.2% for movement disorders specialists and, 76.3% for general neurologists and, 84.6% for residents.

The average onset of SCA3 in our patients was 40±10.74 years, and the disease duration, in years, was 11.86± 6.13. Paulson [4] reported variation of patients with disease onset from 5 to 70 years of age. Schmitz- Hubsch *et al*. [16] found similar data to ours: age of onset of 37.1 ± 11.4 years. In our study, there was no significant correlation when the values of the severity of the dystonia were correlated with the disease SCA3 duration, (r=-0,02852, p=0.9229), or with the onset age of the SCA3 (r=-0,3301, p=0.2491). The variability in the range of values might influence the statistical results.

Reviewing the patients’ data, we could not obtain the onset time of dystonic movements. Difficulty which we believe also occurred with other researchers who proposed to perform similar studies, perhaps because the beginning of the dystonic symptoms in patients with SCA3 has not been regularly addressed, or was justified with a clinical heterogeneity of the SCA3, or even an overlap of symptoms [4, 7, 17, 18].

Despite the phenotype variability, three clinical findings were present in all subjects: gait ataxia, dysarthria and nystagmus; such symptoms were also evaluated as very frequent in SCA3 in several studies [4, 19]. Other ocular symptoms as ophthalmoplegia, pseudoexoftalmia and saccade slow movement of the eyes were present in more than 85% of the patients. All these common symptoms, are important clinical information, easily identifiable by health professionals. This set of symptoms should be familiar to physicians, so that the hereditary ataxias are in their repertoire of diagnosis hypotheses [18, 19].

In agreement with the findings of this study, other non-motor symptoms in SCA3 such as dysphagia, neurogenic bladder, changes in sensitivity, cognitive dysfunction, were described in previous studies [7, 19]. There was no presence, as described in studies as of Schmitz-Hubsch *et al*. [20], of other movement disorders such as myoclonus and chorea.

Our study showed no correlation between the presence of dystonia and the number of CAG repetitions, most likely due to our small sample size and having less than 60% of our patients with available genetic studies. This correlation had been demonstrated in studies of Pedroso *et al*. [19] and Nunes *et al.* [18]. Schmitz-Hubsch *et al*. [16] present correlation between CAG repetition, and early onset dystonia.

The most common MJD-subtype in our sample was the subtype I (71.4%), and the subtype II was the second most prevalent. These findings are consistent with those described in the literature, demonstrating the strong relationship between dystonia and SCA3 subtype I [21]. As expected, subtypes 3, 4, 5, 6 and 7 were not found in patients with dystonia [7]. In our study, there was also no difference in dystonia severity when compared to the patients with subtype 1 and subtype 2 (p=0.4345).

Some authors argue that the correlation between symptoms and subtypes of SCA3 is going in disused [22], perhaps due to genetics that allowed the idea of distinct phenotypes and genotypes in a same disease [19]; or due to the inexorable progression of SCA3, that accumulate symptoms. On the other hand, the phenotypic SCA3 heterogeneity still has much to be explored and discussed [7, 17, 23].

Regarding the classification, the focal dystonia was the most prevalent in our study, and the generalized dystonia was present in 2 patients. These findings were similar to those of Nunes *et al*. [18] whose study evaluated 21 patients being 12 with focal dystonia. The variation of clinical presentation should be considered in the medical evaluations and future scientific studies.

In the present study, there was an important variation of dystonia clinical presentations, including within individuals of the same family. Family number 9 had subjects with generalized dystonia and two others with blepharospasm. Camargo *et al*. [24] demonstrate this possibility also in a family with dystonia DYT6, in which a patient presented segmental clinical signs initiated in adolescence and another, generalized clinical signs with onset on childhood.

The striking heterogeneity that we mentioned is not limited to cases of ataxic dystonia presenting different phenotypes, but also to individuals with SCA3 that do not have ataxia, or that ataxia is not the first symptom. It has been called attention to the importance of focal dystonia isolated as first symptom of SCA3. This situation has already been described by Nunes *et al. [**18**]* with two patients, by Muglan *et al.* [25] with cervical focal dystonia and Mendez-Guerreiro *et al*. [26] whose patient had a writer’s cramp. In our study this also occurred in two patients who were investigated for generalized dystonia, and only with the disease evolution and the diagnosis of another family member with ataxia, SCA3 was confirmed for these patients As happened in this two cases, atypical presentations can lead to an incomplete investigation, or a delay in diagnosis, therefore demonstrating that dystonia can not only happen in isolation, but can also precede, in years, the onset of ataxia [25].

## CONCLUSION

The major conclusions of the study, despite the small sample size, are that dystonia can be unnoticed in clinical practice, the time evolution of the disease, as the widely heterogeneous phenotype of SCA3, can be confounding variables for the diagnosis of SCA3 in patients with dystonia, and dystonia can present in isolation and precede the ataxia onset. Future studies should be designed for a better understanding of the clinic and the pathophysiology of other movement disorders in the SCAs context.

## Figures and Tables

**Table 1:** Clinical and epidemiological characteristics of patients with SCA3 and dystonia.

**Family**	**Patient**	**Gender**	**Age of onset** **(In years)**	**Time of disease** **(In years)**	**Expansions**	**Follow up**
**1**	1	M	30	14	- *	Regular
**2**	2	M	14	15	-	Irregular
	3	M	39	20	20/78	Irregular
	4	M	22	25	-	Irregular
**3**	5	M	23	4	25/77	death
**4**	6	M	28	4	29/76	Irregular
**5**	7	F	24	8	78/23	death
**6**	8	M	30	3	16/77	Irregular
**7**	9	F	17	16	20/75	Missed
**8**	10	M	30	12	22/77	death
**9**	11	F	-	37	-	death
	12	F	20	7	-	death
	13	M	20	13	76/23	regular
	14	M	12	10	-	death

*molecular results from family members (Family 1): maternal aunt (23/60) nephew (14/70)

**Table 2:** Subtype classification of patients with SCA3 and dystonia and their relatives with SCA3.

**Family**	**Patient**	**SCA3 subtype**	**Relatives**		
				**SCA3 subtype**	**Clinical features**
1	1	2	aunt	2	A,D, N
			brother	2	bulging eyes, A,D, N, horizontal and vertical ophthalmoplegia
			nephew	2	A
2	2	1	brother	3	A,D, N, vertical ophthalmoplegia
	3	2	cousin	3	A,D, N, tremor, spasticity, fasciculação corpo, sensib. Face and body fasciculations
	4	2	sister	2	bulging eyes, A,D, N, horizontal ophthalmoplegia, face and body fasciculations, Babinski, hyperreflexia, spasticity
			sister	3	bulging eyes, A,D, N, horizontal, Babinski ophthalmoplegia
			cousin	2	A,D, N, horizontal ophthalmoplegia, Babinski, hyperreflexia
			sister	3	bulging eyes, A,D, N, face and body fasciculations
			brother	3	bulging eyes, A,D, N, horizontal ophthalmoplegia, face and body fasciculations, Babinski,
3	5	1	sister	4	bulging eyes, A,D, N, horizontal ophthalmoplegia, parkinsonism, hyperreflexia, spasticity
			brother	2	A,D, N, horizontal ophthalmoplegia l, hyperreflexia, spasticity
			sister	7	bulging eyes, A,D, N, horizontal ophthalmoplegl, fasciculation, parkinsonism, Babinski, hyperreflexia
4	6	1	brother	2	bulging eyes, A,D, N, horizontal ophthalmoplegia, hyperreflexia
			sister	2	bulging eyes, A,D, N, horizontal ophthalmoplegia, hyperreflexia
			brother	2	bulging eyes, A,D, N, Babinski, hyperreflexia
			father	2	bulging eyes, A,D, N, horizontal ophthalmoplegia, fasciculation, hyperreflexia, esspasticity
			brother	3	bulging eyes, A,D, N, horizontal ophthalmoplegia, fasciculation
			sister	2	A,D, N, hyperreflexia
5	7	1	sister	2	A,D, hyperreflexia
6	8	1	brother	2	bulging eyes, A,D, N, horizontal ophthalmoplegia, fasciculation, hyperreflexia
7	9	1	father	2	bulging eyes, A,D, tremor,Babinski, hyperreflexia, spasticity, fasciculation, neurogenic bladder
			brother	2	bulging eyes, A,D, N, horizontal ophthalmoplegia, hyperreflexia, spasticity, fasciculation
8	10	2	cousin	2	bulging eyes, A,D, N, horizontal ophthalmoplegia, hyperreflexia
			brother	1	A,D, N, horizontal ophthalmoplegia, fasciculation, parkinsonism, hyperreflexia, spasticity
			cousin	3	bulging eyes, A,D, N, horizontal ophthalmoplegia, fasciculation, periferic neuropathy
			cousin	2	A,D, N, hyperreflexia
			cousin	2	A,D, N, horizontal ophthalmoplegia
			cousin	6	A,N, horizontal ophthalmoplegia
			cousin	4	bulging eyes, A,D, N, horizontal ophthalmoplegia, parkinsonism, tremor, fasciculation
9	11	1	-	-	-
	12	1	-	-	-
	13	1	-	-	-
	14	1	-	-	-

A Ataxia, D:Disartria, N: Nystagmus

**Table 3:** Clinical classification and characteristics of dystonia.

Family	Patient	Dystonia Description	MDS Dystonia Classification 2013 [9]		BFM Scale [12]
			**Axel 1:** Clinical Characteristics	**Axel 2:** Etiology	
1	1	Oromandibular (closed) and hands	Young adult, segmenting, progressive, persistent, combined	ND, ADH	21
2	2	Cervical (mild left torticollis)	Adolescent, cervical, progressive, persistent, combined	ND, ADH	1
	3	Hands	Young adults, multifocal progressive, specific action combined	ND, ADH	4
	4	Oromandibular (closed)	Young adult, focal, progressive, persistent, combined	ND, ADH	5
3	5	Oromandibular (closed)	Young adult, focal, progressive, persistent, combined	ND, ADH	5
4	6	Blepharospasm	Young adult, focal, progressive, persistent, combined	ND, ADH	1.5
5	7	Blepharospasm	Young adult, focal, progressive, persistent, combined	ND, ADH	1
6	8	Blepharospasm	Young adult, focal, progressive, persistent, combined	ND, ADH	1.5
7	9	Oromandibular (open)	Adolescent, focal, progressive, persistent, combined	ND, ADH	5
8	10	Hands	Young adults, multifocal progressive, specific action combined	ND, ADH	4
9	11	Blepharospasm	Adolescent, focal, progressive, persistent, combined	ND, ADH	5
	12	Blepharospasm	Young adult, focal, progressive, persistent, combined	ND, ADH	5
	13	Generalized (eyes, mouth, neck, trunk, Upper Limbs)	Adolescent, generalized, progressive, persistent, combined	ND, ADH	4+6+12+6+2+2+1=33
	14	Generalized (eyes, mouth, neck, trunk, Upper Limbs and Lower Limbs)which parts?	Adolescent, generalized, progressive, persistent, combined	ND, ADH	4+6+16+3+4+4+1+1+1= 40

ND: neurodegenerative;ADH: autossomal dominant Hereditary

## LIST OF ABBREVIATIONS

*ATXN3*: = Ataxin
BMF: = Burke Fahn-Marsden scale
CAG: = Cytosine guanine adenosine
HC-UFPR: = Clinical Hospital from Federal University of Paraná
MJD: = Machado Joseph Disease
MSD: = Movement Disorders Society
SCAs: = Spinocerebellar ataxias
SCA3: = Spinocerebellar ataxia type 3
